# Bioactivities of enzymatic protein hydrolysates derived from *Chlorella sorokiniana*

**DOI:** 10.1002/fsn3.1097

**Published:** 2019-06-11

**Authors:** Lhumen A. Tejano, Jose P. Peralta, Encarnacion Emilia S. Yap, Yu‐Wei Chang

**Affiliations:** ^1^ College of Fisheries and Ocean Sciences, Institute of Fish Processing Technology University of the Philippines Visayas Miagao Iloilo Philippines; ^2^ Department of Food Science National Taiwan Ocean University Keelung Taiwan

**Keywords:** angiotensin‐converting enzyme inhibitory, antibacterial activity, antioxidant activity, *Chlorella sorokiniana*

## Abstract

*Chlorella sorokiniana* protein isolates were enzymatically hydrolyzed using pepsin, bromelain, and thermolysin, with their molecular characteristics and bioactivities determined. Thermolysin hydrolysates exhibited the highest degree of hydrolysis (18.08% ± 1.13%). The sodium dodecyl sulfate–polyacrylamide gel electrophoresis (SDS‐PAGE) results showed that peptides with molecular weights <10 kDa were found in the hydrolysates compared to the protein isolates. Bioactivity assays revealed that pepsin peptide fraction <5 kDa showed the highest angiotensin‐converting enzyme (ACE)‐inhibitory (34.29% ± 3.45%) and DPPH radical scavenging activities (48.86% ± 1.95%), while pepsin peptide fraction <10 kDa demonstrated the highest reducing power with 0.2101% ± 0.02% absorbance. Moreover, antibacterial assessment revealed that pepsin hydrolysate and peptide fractions displayed inhibition to the test microorganisms. Overall, the present findings suggest that *C. sorokiniana* protein hydrolysates can be valuable bio‐ingredients with pharmaceutical and nutraceutical application potentials.

## 1 INTRODUCTION

Microalgae can be a good source of valuable nutrients for the growing demands for food and other beneficial substances of the increasing human population. It has received much of the world's attention due to its potential application as a renewable resource and a promising alternative plant protein source (Bleakley & Hayes, 2017; Schwenzfeier, Wierenga, & Gruppen, 2011). It contains potent active compounds for survival, with high industrial application potentials (Becker, 2007; da Silva Vaz, Moreira, de Morais, & Costa, 2016). *Chlorella sorokiniana,* originally named as *C. pyrenoidosa* (Rosenberg et al., 2014), is a freshwater green alga (Waghmare, Salve, LeBlanc, & Arya, 2016). *Chlorella* is considered as one of the most nutritionally and ecologically important microalgae with high protein content value higher than 50%. It is noted as GRAS (generally recognized as safe) product by the USFDA (US Food and Drug Administration) and a good source of protein for the production of protein hydrolysates (Bleakley & Hayes, 2017).

Protein hydrolysates are common intermediate products with easily digestible macronutrients (Mizani, Aminlari, & Khodabandeh, 2005). Among the methods of hydrolysis, enzymatic hydrolysis is the preferred method of producing hydrolysates because it offers milder process conditions and produces higher value products (Kose & Oncel, 2015). Enzymatic hydrolysis generates amino acids and small peptides from the intact proteins, which enhance its nutritive value. Protein hydrolysates are believed to be more effective than intact protein or free amino acids (Wang & Zhang, 2012). Furthermore, it can also improve and change the functional, physiochemical, and/or the sensorial attributes of foods without compromising the nutritive value of the proteins (Choi, Hur, Choi, Konno, & Park, 2009). Aside from food applications, protein hydrolysates can also be used as feed ingredients for animals (Mizani et al., 2005; Silva, Ribeiro, Silva, Cahú, & Bezerra, 2014; Tang, Wu, Zhao, & Pan, 2008). In addition, peptides with certain biological activities had been extracted from various protein sources (Agrawal, Joshi, & Gupta, 2016; Ko, Kim, & Jeon, 2012; Kralovec et al., 2007; Morris et al., 2007; Nikoo, Benjakul, & Xu, 2015; Shih & Cherng, 2012; Wald, Schwarz, Rehbein, Bußmann, & Beermann, 2016). The production of protein hydrolysates might be a promising research direction for microalgal utility.

Possible utilization of microalgae will eventually create new products with great potential to enhance animal growth, treat diseases, and offer healthier food products (Barka & Blecker, 2016; Becker, 2007; da Silva Vaz et al., 2016; Spolaore, Joannis‐Cassan, Duran, & Isambert, 2006). Several literatures have studied the different bioactivities of protein hydrolysates from different microalgae, that is, the anticancer and antibacterial effect of *Chlorella vulgaris* (Sedighi, Jalili, Ranaei‐Siadat, & Amrane, 2016), antioxidant properties of *C. vulgaris* (Sheih, Wu, & Fang, 2009), and ACE‐inhibitory activities of *C. vulgaris* (Sheih, Fang, & Wu, 2009; Suetsuna & Chen, 2001) and from *Spirulina platensis* (Suetsuna & Chen, 2001). However, for *C. sorokiniana*, there are still no reports on the bioactivities of its protein hydrolysate, except for the immunostimulatory effects of its proteins and polysaccharide complexes (Kralovec et al., 2007).

Thus, the aim of the study was to evaluate the bioactivities of protein hydrolysates produced from *C. sorokiniana.* Three commercial enzymes were used to produce the hydrolysates, with their molecular characteristics determined. After fractionation, in vitro bioactivity and stability assays were conducted accordingly.

## 2 METHODS AND MATERIALS

### 2.1 Materials

*Chlorella sorokiniana* was purchased from the Taiwan Chlorella Manufacturing Co., Ltd. Thermolysin (from *Geobacillus stearothermopillus)*, pepsin (from porcine gastric mucosa), and bromelain (from pineapple stem) were obtained from Sigma‐Aldrich. The angiotensin I‐converting enzyme (ACE) from rabbit lung (>2 units/mg) and N‐(3‐[2‐furyl]‐acryloyl)‐phenylalanyl‐glycyl‐glycine (FAPGG) were also purchased from Sigma‐Aldrich. The free radical, 1‐1‐diphenyl‐2‐picrylhydrazyl (DPPH), was acquired from Alfa Aesar. All chemical reagents used were of analytical grade.

### 2.2 Preparation of *C. sorokiniana* protein isolates

Dried *C. sorokiniana* was mixed with distilled water at 1:16 (w/v) ratio. The mixture was pretreated by sonication for 1 hr using a bath sonicator. The pH of the mixture was adjusted to 11.38 by 2 M NaOH for alkaline protein extraction and stirred at 150 rpm for 35 min at room temperature. Centrifugation at 8,750 *g* was done for 35 min to separate the spent biomass from the proteins. Subsequently, the pH of the supernatant was adjusted to 4.01 with 1 M HCl and stirred for 60 min for the isoelectric point precipitation. Lastly, it was centrifuged to collect the solubilized proteins precipitated from the supernatant at 8,750 *g* for 35 min. The protein isolates were lyophilized and stored at −20°C until use (Parimi et al., 2017).

### 2.3 Enzymatic hydrolysis

Enzymatic hydrolyses were conducted by using three commercial enzymes: thermolysin, 65°C at pH 8; pepsin, 37°C at pH 2; and bromelain, 50°C at pH 7. The hydrolysis time and enzyme‐to‐substrate ratio were fixed at 4 hr and 1:100, respectively (Wang & Zhang, 2012). Afterward, the mixtures were heated at 85°C for 15 min to inactivate the enzymes (Morris, Almarales, Carrillo, & Bermúdez, 2008). Then, centrifugation at 8,750 *g* for 15 min was done (Wang & Zhang, 2012). Samples were taken every 30 min for 4 hr for the monitoring of degree of hydrolysis. The hydrolysates were lyophilized and stored at −20°C until used.

#### 2.3.1 Degree of hydrolysis (DH)

O‐phthalic aldehyde (OPA) method as described by Medina, Rubilar, Shene, Torres, and Verdugo (2015) was used to determine the degree of hydrolysis. Briefly, 10 µl of the hydrolysates was mixed with 200 µl of OPA solution (12.5 ml of 100 mM sodium tetraborate, 1.25 ml of 20% SDS, 20 mg OPA in 0.5 ml methanol, and 50 µl b‐mercaptoethanol). Then, the mixture was incubated for 100 s at 37°C. Total acid hydrolysis was performed by adding 6N HCl to the hydrolysates, stirred for 24 hr at 60°C prior to analysis. The absorbance was read at 340 nm using microplate reader (Multiskan GO; Thermo Fisher Scientific). The degree (%) of hydrolysis was calculated using the formula:

$$\text{DH}\left(\%\right)=\{\left[{\left({\text{NH}}_{2}\right)}_{\text{tx}}-{\left({\text{NH}}_{2}\right)}_{\text{t}0}\right]/\left[{\left({\text{NH}}_{2}\right)}_{\text{total}}-{\left({\text{NH}}_{2}\right)}_{\text{t}0}\right]\}\times 100\%,$$

where (NH_2_)_tx_ is the amount of free amino groups at X min, and (NH_2_)_total_ is the amount of total amino groups by total acid hydrolysis. (NH_2_)_t0_ represents the amount of free amino groups at 0 min of hydrolysis.

### 2.4 Sodium dodecyl sulfate–polyacrylamide gel electrophoresis (SDS‐PAGE) analysis

The molecular mass distributions of proteins in the samples were analyzed by SDS‐PAGE (Schägger & Von Jagow, 1987). The protein isolates and hydrolysates were resuspended in denaturant buffer (0.5 M Tris–HCl pH 6.8, glycerol, 10% SDS, w/v, 0.5% bromophenol blue, w/v, β‐mercaptoethanol), at a concentration of 10 mg/ml and heated at 95°C. 10 µl of each sample was loaded onto the 4% stacking gel (w/v) and 12% polyacrylamide gel (w/v). Then, it was run in a Mini‐PROTEAN II unit (Bio‐Rad Laboratories) for 2 hr. Afterward, the gel was stained with Brilliant Blue (Bio‐Rad, Coomassie R250) for 40 min and destained three times using water/methanol/acetic acid (7/2/1, v/v/v), using an orbital shaker (Fristek S10). Thereafter, the gel was scanned with E‐Box VX5 (Vilber Lourmat). The molecular mass of the proteins was measured using a molecular protein mass marker (250 to 10 kDa, Bio‐Rad) loaded at 5 µl in the gel.

### 2.5 Amino acid analysis

The amino acid compositions of the hydrolysates and protein isolates were determined using Hitachi L8900 Amino Acid Analyzer. In a hydrolysis tube, 40 mg of the samples was added with 6 M HCl and then flushed with nitrogen gas for 45 s. Hydrolysis was allowed for 24 hr at 110°C in an oven. After cooling, the mixtures were filtered into 250‐ml round bottom flask. The hydrolysis tube and funnel were washed with 30 ml of deionized water. The filtrates were evaporated to dryness using rotary evaporator. Then, 20 ml of deionized water was added to the dried samples. Afterward, the samples were loaded to the amino acid analyzer.

The free amino acids were separated by ion exchange chromatography and analyzed using Hitachi L‐8900 high‐speed amino acid analyzer, with a Hitachi custom ion exchange resin packed column (4.6 mm ID x 60 mm). Standard lithium buffers were used in the analysis. The absorbance of the amino acid derivatives from postcolumn derivatization with ninhydrin was measured at 570 nm for most amino acids and 440 nm for proline. The levels of free amino acids were estimated on the basis of peak areas of known concentration standards using EZChrom Elite^TM^ chromatography data system. The analyses were done in triplicates.

### 2.6 Fractionation of protein hydrolysates

Fractionation of protein hydrolysates was carried out by ultrafiltration using Lefo Science‐Spectrum Labs MAP‐TFF Systems. The samples were placed in the ultrafiltration hollow fiber membrane with molecular weight cutoffs (MWCOs) of 5 and 10 kDa. The peptide fractions were lyophilized and stored in sealed containers at ‐ 20°C until used. The protein contents of the protein isolates, hydrolysates, and peptide fractions were determined using the modified Lowry method (Markwell, Haas, Bieber, & Tolbert, 1978).

### 2.7 Angiotensin I‐converting enzyme (ACE)‐inhibitory activity assay

The ability of protein hydrolysates and peptide fractions to inhibit the activity of ACE was determined using FAPGG as the synthetic substrate (Lin, Alashi, Aluko, Sun Pan, & Chang, 2017). The samples (1 mg/ml) and 0.5 m/m FAPGG were dissolved in 50 mM Tris‐HCl buffer containing 0.3 NaCl at pH 7.5. Then, 10 µl ACE (0.5 U/ml final activity of 25 mU) was added to 170 µl of FAPGG and 20 µl of sample. The rate of decrease in absorbance at 345 nm was monitored at regular intervals for 30 min at 37°C in a microplate reader (Multiskan Go; Thermo Fisher Scientific). Captopril (1 mg/ml) was used as a positive control, while Tris‐HCl buffer was used as the negative control. ACE activity is expressed as the rate of reaction (ΔA/min), and the inhibitory activity was calculated as follows:

$$\text{ACE}\;\text{inhibition}\;\text{(}\%\text{)}=\left[\frac{\Delta A/\min_{\text{(control)}}-\Delta A/\min_{\left(\text{sample}\right)}}{\Delta A/\min_{\left(\text{control}\right)}}\right]\times 100\%,$$

where $\text{A}/{\text{min}}_{\left(\text{sample}\right)}/\Delta \text{A}/{\text{min}}_{\left(\text{control}\right)}$ are ACE activity in the presence and absence of the peptides, respectively.

### 2.8 Antioxidant properties

#### 2.8.1 DPPH radical scavenging activity assay

The DPPH radical assay was performed according to the method of Girgih, Udenigwe, and Aluko (2011). A 100 µl aliquot of hydrolysates or peptide fractions in methanol (1 mg/ml concentration) was mixed with 100 µl methanolic solution of 0.1 mmol/l DPPH in 96‐well plate. The mixture was left to stand for 30 min in the dark. The absorbance was measured at 517 nm against a blank using a microplate reader (Multiskan Go, Thermo Fisher Scientific). Methanol and ascorbic acid were used as negative and positive control, respectively. The DPPH radical scavenging ability was calculated as follows:

$$\text{DPPH scavenging activity}\;\left(\%\right)=\left[\frac{\left({\text{Abs}}_{\text{control}}{\text{- Abs}}_{\text{sample}}\right)}{{\text{Abs}}_{\text{control}}}\right]\times \text{100}\%.$$

#### 2.8.2 Reducing power assay

The reducing power activity was assayed according to Girgih et al. (2011) with some modifications. The hydrolysates and peptide fractions were dissolved in 0.2 M sodium phosphate buffer (pH 6.6) (1 mg/ml concentration). An aliquot of 250 µl of the solutions was mixed with 250 µl of 1% (w/v) potassium ferricyanide solution and incubated for 30 min at 50 ⁰C. Afterward, 250 µl of 10% (w/v) TCA was added to the mixture and centrifuged at 3,000 rpm for 10 min. The collected supernatant (250 µl) was mixed with 200 µl of distilled water and 50 µl of 0.1% (w/v) ferric chloride. In a 96‐well microplate, 200 µl of the mixture was incubated for 10 min at room temperature and the absorbance was measured at 700 nm using a microplate reader (Multiskan Go; Thermo Fisher Scientific). Distilled water was used as the negative control and ascorbic acid as positive control.

### 2.9 Antibacterial activity assay

The antibacterial properties of the hydrolysates and peptide fractions were conducted by agar well diffusion method, as described by Jemil et al. (2014). Briefly, the protein suspension of 100 mg/ml was sterilized by using 0.22‐nm nylon membrane filter. Cultures of *Staphylococcus aureus and Escherichia Coli* at 1.0 × 10^6^ CFU/ml were spread on tryptone soy agar. Using a sterile borer, wells (8 mm in diameter) were made onto the agar and 50 µl of the protein suspension was seeded into the wells. The plates were placed at 4°C for 1 hr and incubated at 37°C for 24 hr. Penicillin and sterile distilled water were used as positive and negative control, respectively. The antimicrobial activity was evaluated by measuring the diameter of the growth inhibition zone in millimeters.

### 2.10 Statistical analysis

Statistical analysis was performed using SPSS (Statistical Package for Social Science) version 20.0. The significant differences between the mean values for the different tests were determined by one‐way ANOVA and post hoc test by Tukey's test at the level of significance of *p* < 0.05.

## 3 RESULTS AND DISCUSSION

### 3.1 Protein isolation and production of protein hydrolysates

The thick cell wall of the microalgae is one of the biggest challenges in accessing and hydrolyzing its proteins (Cian, Martínez‐Augustin, & Drago, 2012; Kose & Oncel, 2015). This causes low protein digestibility or solubility of the microalgal biomass (Morris et al., 2008). Accordingly, non‐denaturing protein isolation techniques must be employed to extract the protein without compromising its functionality and activity. In this study, alkaline extraction followed by isoelectric point precipitation was utilized to isolate the proteins from the pretreated microalgal biomass. The results showed that the protein extraction process produced an average yield of 4.40% (wt/wt initial biomass dry basis) of the *C. sorokiniana* protein isolates (CSPI), characterized by a protein content of 65.08% (Table 1). The isolate's protein content was higher than *C. sorokiniana* biomass protein which was at 58.23% (Table 1). The yield in the study, 4.40% ± 0.75%, suggests that it is higher than the one reported by Ursu et al. (2014), 2.3% ± 0.2%. In terms of its yield, the combined processes were not sufficient to extract all the microalgal proteins, which may be due to the incomplete disruption of the cell wall of the biomass (Ursu et al., 2014). Furthermore, it was found that the residual supernatant (RSu) after the precipitation process still contained 25.58% protein content. The results imply that some proteins did not precipitate and were not recovered during isolation.

**Table 1:** Degree of hydrolysis, protein contents, and yields of the *C. sorokiniana* protein isolate, hydrolysates, and peptide fractions

Samples	Abbreviations	Protein content (%)	Yield* (%)	Maximum DH (%)
*C. sorokiniana biomass*		58.23 ± 0.35		
Protein isolate	CSPI	65.08 ± 0.88	4.40 ± 0.75	
Pepsin hydrolysate	CSHPe	54.73 ± 0.83^a^	41.27 ± 5.90^a^	8.16 ± 0.29^a^
Bromelain hydrolysate	CSHBr	63.87 ± 0.13^b^	88.55 ± 6.16^b^	15.93 ± 0.77^b^
Thermolysin hydrolysate	CSHTh	84.64 ± 1.51^c^	92.83 ± 10.12^b^	18.08 ± 1.13^b^
Pepsin peptide fraction <5 kDa	HFPe5	65.61 ± 2.26^a^	17.42†	
Pepsin peptide fraction <10 kDa	HFPe10	65.40 ± 5.54^a^	24.09†	
Bromelain peptide fraction <5 kDa	HFBr5	73.84 ± 2.34^a^	15.65†	
Bromelain Peptide Fraction <10 kDa	HFBr10	73.94 ± 3.60^a^	23.72†	
Thermolysin peptide fraction <5 kDa	HFTh5	91.67 ± 1.34^b^	35.13†	
Thermolysin peptide fraction <10 kDa	HFTh10	95.83 ± 2.01^b^	31.63†	

Note

Different superscript letters have significantly different (*p* < 0.05) mean values.

* The yield was calculated based on the dry weight of the lyophilized pellets over the dry weight of the raw material and protein isolate used during isolation and hydrolysis.

^†^ Fractionation was only done once for each fraction of the hydrolysates; thus, no replicate was reported for the yields of the fractions.

**Figure 1:**
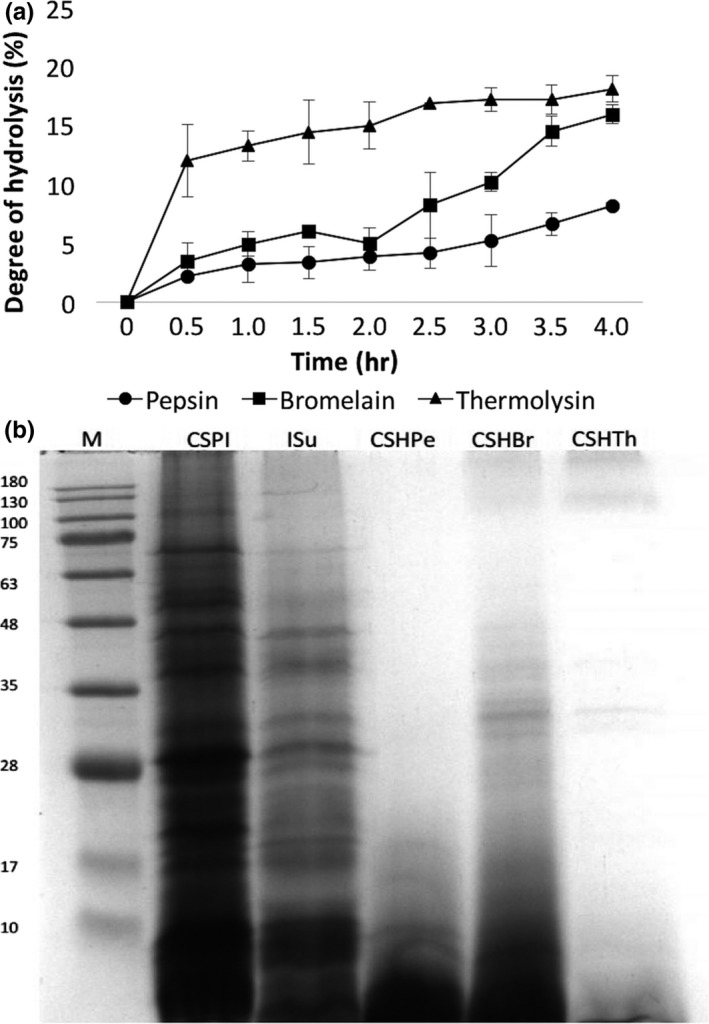
(a) Degree of hydrolysis of *C. sorokiniana* protein isolates during 4 hr of enzymatic hydrolysis using pepsin, bromelain, and thermolysin, and (b) SDS‐PAGE of *C. sorokiniana* protein isolates (CSPI), residual supernatant (RSu), and *C. sorokiniana* protein hydrolysates by the following: pepsin (CSHPe), bromelain (CSHBr), and thermolysin (CSHTh)

Subsequently, the protein isolates were hydrolyzed by three commercial enzymes to evaluate their biological properties. The results revealed that thermolysin hydrolysates (CSHTh) have the highest degree of hydrolysis (DH) of 18.08%, followed by bromelain hydrolysates (CSHBr) with 15.93%, and, finally, pepsin hydrolysates (CSHPe) with 8.16% after 4 hr of hydrolysis (Figure 1a). CSHPe showed significantly lowest DH among the hydrolysates at *p* < 0.05. The results showed that the hydrolyses gradually increased over time, which were in agreement with the results reported by Morris et al. (2008) for *Chlorella* proteins, and X. Wang and Zhang (2012) for *Chlorella pyrenoidosa.* In this study, DH of CSHTh and CSHBr was higher than 10%. According to Morris et al. (2008), achieving this level, the processed hydrolysates have the potential to be used for pharmaceutical products.

### 3.2 Distribution of proteins/peptides

Gel electrophoresis was employed to observe the distribution of the proteins and estimate their molecular masses (Ursu et al., 2014). The results showed that the proteins present in CSPI were highly diverse (Figure 1b), which may be elucidated by the fact that microalgae do not have specific protein storage as their nitrogen source (Schwenzfeier et al., 2011). The protein distribution in RSu resembles those of the CSPI. The proteins in the RSu were the same proteins that were not precipitated and remained in the solution after hydrolysis. In addition, CSPI showed more distinct bands than RSu, as only small amounts of those proteins remained in the RSu after isolation. Furthermore, most of the proteins found in the CSPI and RSu could be proteins/enzymes, that is, cell wall structural proteins, cell wall‐modifying enzymes, flagellum‐related proteins, heat‐shock proteins, transport/binding proteins/lipoproteins, and photosynthetic enzymes (Contreras et al., 2008; Wang, Hu, Sommerfeld, & Chen, 2004). These proteins could have multiple polypeptide chains as shown in SDS‐PAGE results (Schwenzfeier et al., 2011).

The protein profiles of the three hydrolysates showed that its contents are mostly smaller peptides of ≤10 kDa. In addition, the resulting hydrolysates have mixed peptides, only in small quantities; as shown in the gel having no distinct protein bands. The results affirmed that enzymatic hydrolysis was effective in producing low molecular mass compounds of <10 kDa, a result favorable to expect potent bioactivity of the peptides (Medina et al., 2015; Sarmadi & Ismail, 2010).

### 3.3 Amino acid profiles

The presence of the essential amino acids greatly determines the quality and nutritive value of proteins to meet the requirements for animal body functions (Brown, Jeffrey, Volkman, & Dunstan, 1997; Yücetepe & Özçelik, 2016). It is observed in Table 2 that the amino acid profiles of the protein isolates and hydrolysates were almost identical, suggesting that their protein quality is similar (Brown et al., 1997). In addition, the abundance of all essential amino acids, except for tryptophan, was observed in all samples. Aspartic acid, glycine, alanine, and proline were the most abundant amino acids present in all samples, with glutamic acid values as the highest. The results were comparable to the reports of Ursu et al. (2014) and Morris et al. (2008) on *C. vulgaris*. The results were also supported by the findings of Brown et al. (1997) on 40 different species of microalgae having almost similar amino acid profiles.

**Table 2:** Amino acid profiles and comparative study of essential amino acid patterns with FAO/WHO/UNU standard of *C. sorokiniana* protein isolate (CSPI); and *C. sorokiniana* protein hydrolysates by pepsin (CSHPe), bromelain (CSHBr), and thermolysin (CSHTh)

Amino acids (g/100 g protein)	CSPI	CSHBr	CSHPe	CSHTh	WHO/FAO (2007)	
					Children (3–10 years)	Adult
Histidine^a^	2.03 ± 0.24	1.82 ± 0.68	1.68 ± 0.68	2.00 ± 0.35	1.6	1.5
Isoleucine^a^	3.33 ± 0.80	2.66 ± 1.18	2.33 ± 0.91	3.06 ± 0.22	3.1	3.0
Leucine^a^	10.25 ± 1.24	9.20 ± 1.58	7.62 ± 2.62	9.72 ± 1.11	6.1	5.9
Lysine^a^	7.70 ± 3.48	6.48 ± 0.63	6.49 ± 0.32	7.88 ± 3.02	4.8	4.5
Threonine^a^	4.49 ± 1.05	4.49 ± 1.84	4.49 ± 1.84	5.13 ± 1.73	2.5	2.3
Valine^a^	8.22 ± 1.31	6.75 ± 2.72	8.35 ± 3.93	9.39 ± 2.43	4.0	3.9
Met^a^ + Cys	5.35 ± 0.38	4.75 ± 0.93	4.37 ± 0.99	5.42 ± 0.51	2.4	2.2
Phe^a^ + Try	9.84 ± 1.26	8.14 ± 2.82	7.38 ± 2.42	9.81 ± 1.20	4.1	3.8
Arginine^a^	8.04 ± 1.18	7.47 ± 2.80	6.66 ± 2.72	8.01 ± 1.63		
Serine	5.35 ± 1.12	4.71 ± 0.94	4.34 ± 1.09	7.23 ± 2.79		
Glutamic acid	18.73 ± 2.74	15.52 ± 6.06	15.48 ± 5.60	17.61 ± 1.78		
Glycine	8.90 ± 0.64	7.77 ± 2.47	7.44 ± 1.92	10.25 ± 0.45		
Alanine	11.74 ± 1.44	9.80 ± 3.64	10.58 ± 2.53	11.12 ± 1.11		
Aspartic acid	13.96 ± 1.97	7.54 ± 1.63	6.90 ± 1.71	6.04 ± 1.95		
2‐aminoethanol	0.43 ± 0.05	0.25 ± 0.08	0.03 ± 0.01	0.37 ± 0.03		
Ammonia	2.20 ± 0.64	2.18 ± 1.10	1.15 ± 0.29	2.18 ± 0.70		
Ornithine	0.25 ± 0.14	0.18 ± 0.08	0.25 ± 0.11	0.20 ± 0.04		
Hydroxyproline	14.12 ± 5.36	14.94 ± 1.17	11.90 ± 0.52	10.37 ± 2.11		
Proline	17.27 ± 0.01	15.43 ± 6.32	14.40 ± 4.13	18.53 ± 0.12		

Note

The values are the mean ± *SD* of three independent determinations.

a Essential amino acids.

The results revealed that glutamic acid and aspartic acid were present in high amounts, which is true to most microalgae (Medina et al., 2015). In the current study, lysine, considered as a limiting amino acid in cereals (Brown et al., 1997; Morris et al., 2008), was abundant in the isolates and hydrolysates. Its abundance suggests that the isolate and hydrolysates can be used as a suitable substitute or an ingredient for foods or food blends. Furthermore, in comparison with the WHO/FAO/UNU (World Health Organization & University, 2007) amino acid requirement for children (3–10 years) and adults, *C. sorokiniana* protein isolate and the hydrolysates showed greater values than the reference values shown in Table 2. This indicates that the protein isolates and hydrolysates from *C. sorokiniana* could be an excellent potential source of protein to meet protein requirements for both children and adults.

### 3.4 ACE‐inhibitory activity

**Figure 2:**
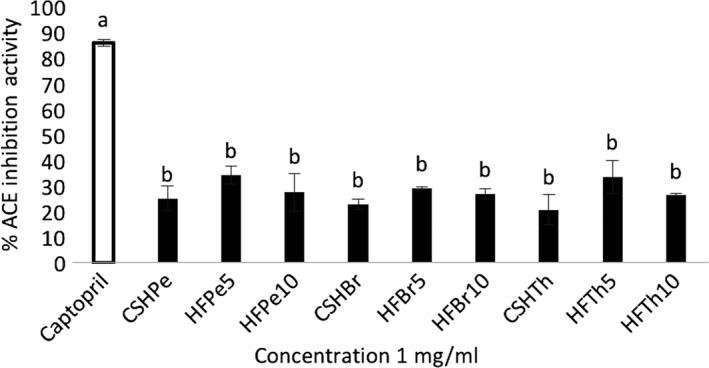
In vitro ACE inhibition activities of *C. Sorokiniana* protein hydrolysates by the following: pepsin (CSHPe), bromelain (CSHBr), and thermolysin (CSHTh); and protein fractions: pepsin fraction <5 kDa (HFPe5), pepsin fraction <10 kDa (HFPe10), bromelain fraction <5 kDa (HFBr5), bromelain fraction <10 kDa (HFBr10), thermolysin fraction <5 kDa (HFTh5), and thermolysin fraction <10 kDa (HFTh10). Bars with different letters are significantly different at *p* < 0.05

After fractionation, the potential bioactivities of the protein hydrolysates and peptide fractions were investigated including their ACE‐inhibitory effects. As shown in Figure 2, all the protein hydrolysates and peptide fractions exhibited ACE‐inhibitory activities. The results demonstrated that there are ACE‐inhibitory peptides encrypted in *C. sorokiniana* proteins. Results further revealed that the <5 kDa pepsin peptide fraction (HFPe5) showed the highest ACE‐inhibitory activity (34.29% ± 3.45%). On the other hand, CSHTh exhibited the lowest ACE‐inhibitory activity. The peptide fractions displayed higher ACE‐inhibitory activities than their protein hydrolysate counterparts, with peptide fractions <5 kDa showing the greatest activities. This showed that fractionation was effective in increasing the ACE‐inhibitory activity of the hydrolysates. However, no significant differences (*p* < 0.05) were found among the samples.

Pepsin hydrolysates from *C. vulgaris* protein waste (Sheih, Fang, et al., 2009), *C. vulgaris* biomass (Suetsuna & Chen, 2001), and *S. platensis* biomass (Suetsuna & Chen, 2001) were also reported to have high ACE‐inhibitory activities. Moreover, several researches reported the effectiveness of pepsin to produce hydrolysates and peptide fractions with high ACE‐inhibitory activity from dried bonito (Yokoyama, Chiba, & Yoshikawa, 1992), from tilapia frame (Lin et al., 2017), and from flaxseed (Udenigwe, Lin, Hou, & Aluko, 2009). Pepsin may have cleaved the hydrophobic residues of the polypeptide chains in the hydrolysates leading to exhibit more ACE‐inhibitory activity (Hong et al., 2008). As to the knowledge of the researchers, there are no reports on the ACE‐inhibitory activities of hydrolysates from microalgae using bromelain and thermolysin. However, for macroalgae, Paiva, Lima, Neto, and Baptista (2016) stated that bromelain hydrolysate from *Ulva rigida* showed the highest ACE‐inhibitory activity among other hydrolysates in the study. Moreover, Ghanbari et al. (2015) accounted that the bromelain hydrolysate from sea cucumber exhibited one of the highest ACE‐inhibitory activities among other hydrolysates produced using various proteases. On the other hand, thermolysin was reported to produce a digest from dried bonito which exhibited antihypertensive effects (Yokoyama et al., 1992). Nonetheless, the results demonstrated that protein hydrolyses using the three enzymes were able to release ACE‐inhibitory peptides from microalgal proteins.

In the current study, the hydrolysates were found to have high amounts of hydrophobic amino acids such as phenylalanine, tyrosine, and proline, which are reported to exhibit potent ACE‐inhibitory activity when positioned at the C‐terminal side of the peptides (Kim & Chung, 1999; Kohmura et al., 1989; Li, Matsui, Matsumoto, Yamasaki, & Kawasaki, 2002; Maruyama et al., 1987; Miyoshi et al., 1991). The abundance of these amino acids in the hydrolysates and fractions may have greatly contributed to their ACE‐inhibitory activities.

### 3.5 Antioxidant properties

#### 3.5.1 DPPH radical scavenging activity

**Figure 3:**
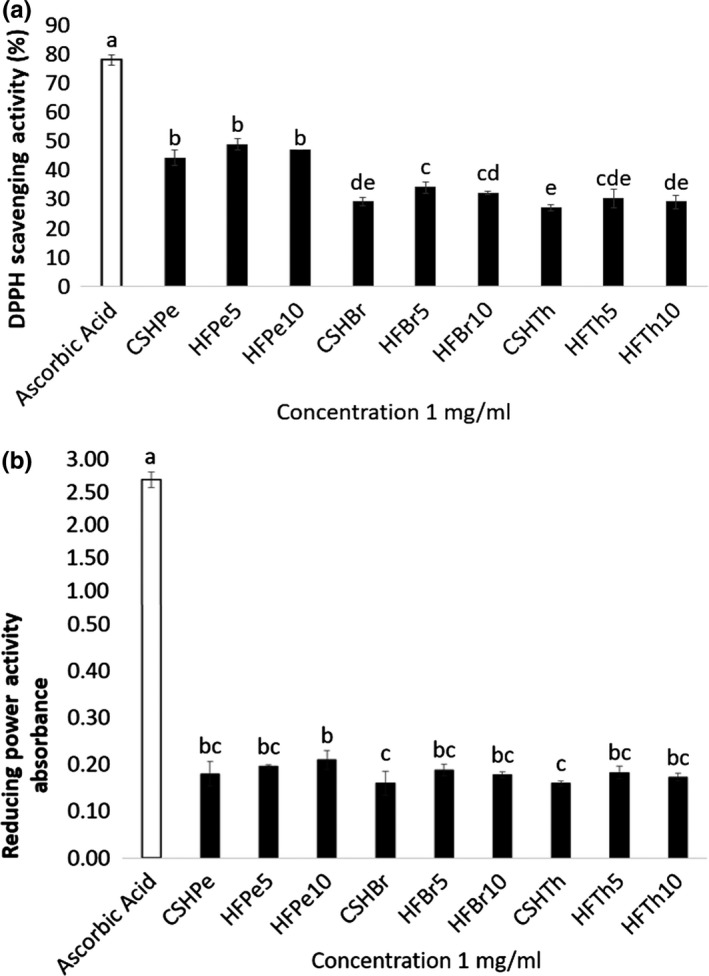
(a) In vitro DPPH radical scavenging activities and (b) reducing power activities of *C. Sorokiniana* protein hydrolysates by the following: pepsin (CSHPe), bromelain (CSHBr), and thermolysin (CSHTh); and protein fractions: pepsin fraction <5 kDa (HFPe5), pepsin fraction <10 kDa (HFPe10), bromelain fraction <5 kDa (HFBr5), bromelain fraction <10 kDa (HFBr10), thermolysin fraction <5 kDa (HFTh5), and thermolysin fraction <10 kDa (HFTh10). Bars with different letters are significantly different at *p* < 0.05

The DPPH radical scavenging activities (RSA) of the protein hydrolysates and peptide fractions from *C. sorokiniana* were also determined in the study. The results revealed that HFPe5 exhibited the highest DPPH RSA (48.86% ± 1.95%). It was closely followed by pepsin peptide fraction <10 kDa (HFPe10) with 47.12% ± 0.06% DPPH RSA (Figure 3a). CSHTh and CSHBr showed low DPPH radical scavenging activities of 27.12% ± 0.92% and 29.10% ± 1.43%, respectively. Peptide fractions with lower molecular weights were found to have higher DPPH RSA than their protein hydrolysate counterparts, which were in agreement with the previous reports of Girgih et al. (2011) and Chalamaiah, Jyothirmayi, Diwan, and Kumar (2015). In most cases, significant differences (*p* < 0.05) were observed in the DPPH RSA of the protein hydrolysates and peptide fractions. Pepsin hydrolysate and peptide fractions showed significantly higher activities than other samples, which were in accordance with the results of Ko et al. (2012).

Peptide's molecular structure and weight affect their antioxidant properties (Sheih, Wu, et al., 2009). Furthermore, their amino acid sequence and composition may contribute to their higher antioxidant properties (Chalamaiah, Hemalatha, et al., 2015). Aromatic amino acids such as tryptophan and tyrosine are reported to have strong antioxidant effects (Ko et al., 2012). By direct electron transfer, these aromatic residues can easily cause oxygen to be stable. In addition, hydrophobic amino acid residues also influence the antioxidant properties of peptides (Ko et al., 2012). The amino acid profile of the hydrolysates revealed abundance of hydrophobic amino acids which may have influenced their DPPH RSA. In general, the protein hydrolysates and peptide fractions showed good electron donating capabilities, thus scavenging DPPH radicals.

#### 3.5.2 Reducing power activity

As observed in Figure 3b, the reducing power of the protein hydrolysates and peptide fractions was varied. The highest reducing power activity was exhibited by HFPe10 with 0.2101 ± 0.02 absorbance at 700 nm wavelength. It was closely followed by HFPe5 with 0.1958 ± 0.003 absorbance. The results were in agreement with the results reported by Girgih et al. (2011), wherein larger peptide fractions obtained from hemp seed hydrolysates showed better reducing power than smaller peptide fractions. No significant differences at *p* < 0.05 were found in the reducing power activities of samples in the current study. Though lower than the standard used, the results showed that the protein hydrolysates and peptide fractions have the ability to donate electron and act as reducing agents. The results were comparable to the observed reducing power activities at 1mg/ml concentration of the following: hemp seed protein hydrolysate at <0.15 absorbance (Girgih et al., 2011) and fish meats fermented by *Bacillus subtilis* at <0.2 absorbance (Jemil et al., 2014).

Peptides showing high absorbance demonstrate higher reducing power caused by the ability to donate hydrogen or electron (Chalamaiah, Jyothirmayi, et al., 2015). The increased availability of hydrogen ions may have contributed to the strong reducing power of the hydrolysates. In addition, the presence of amino acids such as histidine, leucine, isoleucine, lysine, tyrosine, and methionine in the protein hydrolysates and peptide fractions may have influenced their reducing power activities (Girgih et al., 2011).

The results suggest that the *C. sorokiniana* protein hydrolysates and peptide fractions might have some reductones and can be used as reducing agents. The varying reducing powers of the protein hydrolysates and peptide fractions may have been influenced by varying proteases used in the hydrolysis, which could produce peptides with varying compositions, sequences, and sizes (Chalamaiah, Jyothirmayi, et al., 2015).

### 3.6 Antibacterial activity

The antibacterial properties of the protein hydrolysates and peptide fractions were evaluated against *E. coli* and *S. aureus*. Table 3 presents the results using agar well diffusion method. It can be observed that only CSHPe, HFPe5, and HFPe10 had exhibited antibacterial activities against test microorganisms. In addition, the growth inhibition zone diameters of the peptide fractions were larger than that of the hydrolysate. This indicates the higher activity of the low molecular weight peptides compared to that of the large compounds in the hydrolysates. The results were in agreement with Sedighi et al. (2016), wherein low molecular weight peptides from *Chlorella sp.* hydrolysates effectively induced the destruction of the *E. coli* cell wall as shown by higher inhibition than the microalgal biomass. All other protein hydrolysates and peptide fractions showed no inhibition effect to the test microorganisms.

**Table 3:** Antibacterial activities of *C. sorokiniana* protein hydrolysates and peptide fractions

Samples	Growth Inhibition Zone Diameter (GIZD)*	
	*Staphylococcus aureus*	*Escherichia coli*
CSHPe	15 ± 1.0	13 ± 1.0
HFPe5	15 ± 2.0	17 ± 2.0
HFPe10	25 ± 4.0	14 ± 1.0
CSHBr	−	−
HFBr5	−	−
HFBr10	−	−
CSHTh	−	−
HFTh5	−	−
HFTh10	−	−
Penicillin	+	27 ± 2.0

Note

*C. Sorokiniana* protein hydrolysate by the following: pepsin (CSHPe), bromelain (CSHBr) and thermolysin (CSHTh); and protein fractions: pepsin fraction <5 kDa (HFPe5), pepsin fraction <10 kDa (HFPe10), bromelain fraction <5 kDa (HFBr5), bromelain fraction <10 kDa (HFBr10), thermolysin fraction <5 kDa (HFTh5), and thermolysin fraction <10 kDa (HFTh10).

−, no inhibition detected; +, optimum inhibition

* The values (in millimeters) represent averages ± standard deviations for triplicate experiments (*n* = 3).

It is also noteworthy to mention that the pepsin hydrolysate and peptide fractions had more inhibition effect to gram‐positive bacteria, *S. aureus*. The results were in concurrence to previous studies which stated that gram‐negative bacteria are more resistant to antimicrobial substances than gram‐positive bacteria because of their cell wall (Lambert, Skandamis, Coote, & Nychas, 2001; Ördög et al., 2004). Their multilayered cell walls hinder the deep penetration of antimicrobials in the cell (Ördög et al., 2004). Interestingly, the inhibition against *S. aureus* of pepsin protein hydrolysate and peptide fractions was particularly of great importance as this microorganism is known to be resistant to a number of phytochemicals (Chakraborty, Mahapatra, & Roy, 2011; Thaker, Brahmbhatt, Nayak, & Thaker, 2013). The antibacterial activities of AMPs are affected by its helical structure, size, charge, and hydrophobicity. Hydrophobic property is necessary in the separation of peptides in the membrane (Tossi, Sandri, & Giangaspero, 2000). Moreover, the cationic property replaces native divalent cations from the charged lipopolysaccharides (LPS) disturbing the outer membrane and weakening the integrity of the cytoplasmic membrane, which results in the penetration of the peptide in the cytoplasm (Powers & Hancock, 2003). Furthermore, antimicrobial activities are influenced by the algal species, peptides extraction method, and the test microorganisms’ resistance (Al‐Saif, Abdel‐Raouf, El‐Wazanani, & Aref, 2014).

## 4 CONCLUSIONS

Protein hydrolysates prepared from *C. sorokiniana* using plant, animal, and bacterial enzymes exhibited varying degree of hydrolysis and molecular characteristics. The hydrolysates have high nutritional value as reflected by their protein contents and amino acid profiles. In addition, the hydrolysates and peptide fractions demonstrated varying bioactivities. In this case, higher DH did not necessarily correlate to higher bioactivity. Pepsin hydrolysate and peptide fractions showed higher ACE‐inhibitory, DPPH scavenging radical, reducing power and antibacterial activities. Though, fractionation increased the activities of the hydrolysates, only in DPPH scavenging radical activity did the hydrolysates and peptide fractions show significant differences. Further fractionation did not significantly increase the ACE inhibition and reducing power activities of the microalgal proteins. These findings suggest that high‐value protein hydrolysates and peptide fractions derived from *C. sorokiniana* have interesting characteristics and bioactivities that may find potential pharmaceutical and/or food applications, providing alternative use of microalgae which adds to its value. Conversely, in vivo analyses and safety must be conducted prior to therapeutic use of the products.

## CONFLICTS OF INTEREST

There are no conflicts of interest.

## ETHICAL APPROVAL

This report does not conduct any human or animal tests.
